# External validation of SAPS 3 and MPM_0_-III scores in 48,816 patients from 72 Brazilian ICUs

**DOI:** 10.1186/s13613-017-0276-3

**Published:** 2017-05-18

**Authors:** Giulliana Martines Moralez, Ligia Sarmet Cunha Farah Rabello, Thiago Costa Lisboa, Mariza da Fonte Andrade Lima, Rodrigo Marques Hatum, Fernando Vinicius Cesar De Marco, Alessandra Alves, Jorge Eduardo da Silva Soares Pinto, Hélia Beatriz Nunes de Araújo, Grazielle Viana Ramos, Aline Reis Silva, Guilherme Côrtes Fernandes, Guilherme Brenande Alves Faria, Ciro Leite Mendes, Roberto Álvaro Ramos Filho, Valdênia Pereira de Souza, Pedro Emmanuel Alvarenga Americano do Brasil, Fernando Augusto Bozza, Jorge Ibrain Figueira Salluh, Marcio Soares, On Behalf of the ORCHESTRA Study Investigators

**Affiliations:** 1 Graduate Program in Translational Medicine, D’Or Institute for Research and Education (IDOR), Rio de Janeiro, Brazil; 2 PPG Internal Medicine, Federal University of Rio de Janeiro, Rio de Janeiro, Brazil; 3 ICU, Hospital Copa D’Or, Rio de Janeiro, Brazil; 4 Complexo Hospitalar, Santa Casa de Misericórdia de Porto Alegre, Porto Alegre, Brazil; 5 ICU, Hospital Esperança Recife, Recife, Brazil; 6 ICU, Hospital Total Cor, Rio de Janeiro, Brazil; 7 ICU, Hospital viValle, São José dos Campos, Brazil; 8 ICU, Hospital Rios D’Or, Rio de Janeiro, Brazil; 9 ICU, Hospital Norte D’Or, Rio de Janeiro, Brazil; 10 Hospital do Coração do Brasil, Brasília, Brazil; 11 Department of Critical Care, D’Or Institute for Research and Education (IDOR), Rio de Janeiro, Brazil; 12 ICU, Santa Casa de Misericórdia de Juiz de Fora, Juiz de Fora, Brazil; 13 ICU, Hospital Oeste D’Or, Rio de Janeiro, Brazil; 14 ICU, Hospital Universitário Lauro Wanderley, João Pessoa, Brazil; 15 ICU, Hospital São Luiz – Unidade Jabaquara, São Paulo, Brazil; 16 Complexo Hospitalar de Niterói, Niterói, Brazil; 17 Instituto Nacional de Infectologia Evandro Chagas, Fundação Oswaldo Cruz-Fiocruz, Rio de Janeiro, Brazil; 18 Department of Critical Care and Graduate Program in Translational Medicine, D’Or Institute for Research and Education (IDOR), Rio de Janeiro, Brazil; 19 Department of Critical Care, D’Or Institute for Research and Education, Rua Diniz Cordeiro, 30. Botafogo, Rio de Janeiro, 22281-100 Brazil

**Keywords:** Severity-of-illness scores, Validation, Intensive care units, Outcomes, Standardized mortality rate

## Abstract

**Background:**

The performance of severity-of-illness scores varies in different scenarios and must be validated prior of being used in a specific settings and geographic regions. Moreover, models’ calibration may deteriorate overtime and performance of such instruments should be reassessed regularly. Therefore, we aimed at to validate the SAPS 3 in a large contemporary cohort of patients admitted to Brazilian ICUs. In addition, we also compared the performance of the SAPS 3 with the MPM_0_-III.

**Methods:**

This is a retrospective cohort study in which 48,816 (medical admissions = 67.9%) adult patients are admitted to 72 Brazilian ICUs during 2013. We evaluated models’ discrimination using the area under the receiver operating characteristic curve (AUROC). We applied the calibration belt to evaluate the agreement between observed and expected mortality rates (calibration).

**Results:**

Mean SAPS 3 score was 44.3 ± 15.4 points. ICU and hospital mortality rates were 11.0 and 16.5%. We estimated predicted mortality using both standard (SE) and Central and South American (CSA) customized equations. Predicted mortality rates were 16.4 ± 19.3% (SAPS 3-SE), 21.7 ± 23.2% (SAPS 3-CSA) and 14.3 ± 14.0% (MPM_0_-III). Standardized mortality ratios (SMR) obtained for each model were: 1.00 (95% CI, 0.98–0.102) for the SAPS 3-SE, 0.75 (0.74–0.77) for the SAPS 3-CSA and 1.15 (1.13–1.18) for the MPM_0_-III. Discrimination was better for SAPS 3 models (AUROC = 0.85) than for MPM_0_-III (AUROC = 0.80) (*p* < 0.001). We applied the calibration belt to evaluate the agreement between observed and expected mortality rates (calibration): the SAPS 3-CSA overestimated mortality throughout all risk classes while the MPM_0_-III underestimated it uniformly. The SAPS 3-SE did not show relevant deviations from ideal calibration.

**Conclusions:**

In a large contemporary database, the SAPS 3-SE was accurate in predicting outcomes, supporting its use for performance evaluation and benchmarking in Brazilian ICUs.

**Electronic supplementary material:**

The online version of this article (doi:10.1186/s13613-017-0276-3) contains supplementary material, which is available to authorized users.

## Background

Severity-of-illness scores have broad applicability in intensive care setting. Although they should not be used on individual basis, they are useful to evaluate ICU performance, to monitor it overtime, to guide resource management and quality improvements, and for benchmarking purposes [1]. However, the performance of these models varies in different scenarios because of differences in case mix, clinical management patterns, admission policies as well as pre- and post-ICU care. Therefore, severity-of-illness scores must be validated prior to their use in a specific setting or geographic region.

The three most used severity-of-illness scores are the Acute Physiology and Chronic Health Evaluation (APACHE) [2], the Mortality Probability Models (MPM_0_-III) [3] and the Simplified Acute Physiology Score (SAPS 3) [4, 5]. Among them, the only score developed using data from patients and intensive care units (ICU) worldwide (307 ICUs in 35 countries) was the SAPS 3 score. Besides a general standard equation, investigators also developed seven regional equations to estimate hospital mortality, thus allowing comparisons among ICUs on a more common level.

In 2009, the Brazilian Association of Intensive Care (Associação de Medicina Intensiva Brasileira, AMIB) chose the SAPS 3 score as the severity-of-illness score recommended for performance evaluation and benchmarking in Brazilian ICUs [6]. However, to our knowledge, validation studies reported conflicting results and were mostly single centered, involving specific patient populations [7–13] and with relatively small sample sizes [14–16]. Moreover, as the calibration of severity-of-illness scores is expected to deteriorate overtime, the performance of such instruments should be reassessed on a regular basis [17]. Therefore, in the present study, we aimed at to validate the SAPS 3 in a large contemporary cohort of patients admitted to Brazilian ICUs. In addition, we also compared the performance of the SAPS 3 with the MPM_0_-III.

## Methods

### Design and setting

This was a secondary analysis of the ORCHESTRA study, a multicenter retrospective cohort study of critical care organization and outcomes in 59,693 patients admitted to 78 ICUs at 51 Brazilian hospitals during 2013 [18].

### Selection of participants, data collection and definitions

**Fig. 1:**
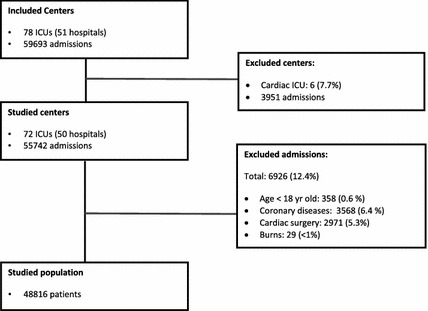
Study flowchart

Participating ICUs in the ORCHESTRA study were selected from the Brazilian Research in Intensive Care Network (BRICNet). For the purposes of the present study, we excluded ICUs exclusively admitting cardiac patients (*n* = 6) (Fig. 1) and a total of 72 ICUs at 50 hospitals were involved. We included all consecutive patients aged ≥16 years admitted to the participating ICUs during 2013. In the ORCHESTRA study, readmissions and patients with missing core data [age, location before ICU admission, main ICU admission diagnosis, SAPS 3 score, ICU and hospital length of stay (LOS) and vital status at hospital discharge] were excluded. In the present study, besides the patients admitted to cardiac units (*n* = 3951), we also excluded those who did not meet both the SAPS 3 and MPM_0_-III eligibility criteria [patients aged <18 years (*n* = 358), who underwent cardiac surgeries (*n* = 2971), with acute myocardial infarction (*n* = 3568) and burns (*n* = 29)]. Therefore, a total of 48,816 patients constituted the study population.

We obtained de-identified patient data from the Epimed Monitor System^®^, (Epimed Solutions^®^, Rio de Janeiro, Brazil), a commercial cloud-based registry for quality improvement, performance evaluation and benchmarking purposes. ICUs using the Epimed Monitor System^®^ prospectively collect data in a structured electronic case report form, most typically using a trained case manager. Key data elements included demographics, admission diagnosis, location before ICU admission, comorbidities based on the Charlson Comorbidity Index [19], functional status one week before hospital admission [20], scores including the SAPS 3 score, MPM_0_-III score and the Sequential Organ Failure Score (SOFA) [21], use of ICU support, ICU and hospital LOS and destination after hospital discharge. The SAPS 3 and MPM_0_-III scores were calculated using data from the ICU admission (±1 h). As recommended, missing values were coded as the reference or “normal” category for each variable. Estimated mortality rates using both the standard equation (SAPS 3-SE) and the one customized for Central and South American countries (SAPS 3-CSA) are provided in the system. In the present study, the primary outcome of interest was in-hospital mortality at the patient level.

### Statistical analysis

We described ICU and patient characteristics using standard descriptive statistics and reported continuous variables as mean ± standard deviation or median (25–75% interquartile range, IQR), as appropriate. We reported categorical variables as absolute numbers (frequency percentages).

We assessed models’ discrimination (ability of each model to discriminate between patients who lived from those who died) by estimating the area under the receiver operating characteristic curve (AUROC). Comparisons between AUROCs by a pairwise evaluation of the three scores discrimination power were performed by Delong method [22]. We used the calibration belt, proposed by the GiViTI group [23, 24], to investigate the relationship between the observed and expected outcomes. Using this approach, a generalized polynomial logistic function between the outcome and the logit transformation of the predicted probability was fitted, with the respective 95 and 80% confidence intervals (CI) boundaries. A statistically significant deviation from the bisector (the line of perfect calibration) occurs when the 95% CI boundaries of the calibration belt do not include the bisector [23]. Calibration curves were constructed by plotting predicted mortality rates (*x*-axis) against observed mortality rates (*y*-axis). Standardized mortality rates (SMR) with respective 95% confidence intervals (CI) were calculated for each model by dividing observed by predicted mortality rates. A two-tailed *p* value <0.05 was considered statistically significant. We performed the statistical analyses using R (http://www.r-project.org) and SPSS 21 (IBM Corp., Armonk, NY).

## Results

Our final sample consisted of 48,816 patients admitted by 72 ICUs in the study period (Fig. 1). Table 1 gives the main hospital and ICU characteristics. Most of ICUs were medical–surgical (*n* = 62, 86.1%) located at private hospitals (*n* = 45, 90.0%). Median number of patients per ICU was 517 (361–817).

**Table 1:** Hospital and ICU characteristics

Characteristics	
Hospitals	(*n* = 50)
Type of hospital	
Private, for profit	31 (62.0%)
Private, philanthropic	14 (28.0%)
Public	5 (10.0%)
Hospital beds (*n*)	
<150	19 (38.0%)
150–300	20 (40.0%)
≥301	11 (22.0%)
Intermediate/step-down unit	
No	25 (50.0%)
Yes	25 (50.0%)
Training programs in critical care	
No	28 (56.0%)
Yes	22 (44.0%)

ICUs	(*n* = 72)
Medical–surgical ICU	
Yes	62 (86.1%)
No	10 (13.9%)
Active ICU beds (*n*)	22 (11–34)
≤10	23 (31.9%)
10–20	27 (37.5%)
>20	22 (30.6%)
ICU bed occupancy rate (%)	73 (62–83)

Results for continuous variables are reported as mean ± SD and median (IQR)

*ICU* intensive care unit, *IQR* interquartile range, *SD* standard deviation

Table 2 reports the main patients’ characteristics. The main reasons for ICU admission were postoperative care (26.3%), followed by sepsis (22.3%), cardiovascular complications (11.3%) and neurological complications (11.4%). At ICU admission, invasive mechanical ventilation was used in 7550 (15.5%) and noninvasive ventilation was used in 4875 (10.0%) of patients. Vasopressors were required by 6158 (12.6%) and renal replacement therapy by 1578 (3.2%).

**Table 2:** Main patients’ characteristics and outcomes

Characteristics	
Patients (*n*)	48,816
Age (years)	65 (48–79)
<45	10,354 (21.2%)
45–64	13,555 (27.8%)
65–74	8661 (17.7%)
75–84	9666 (19.8%)
≥85	6580 (13.5%)
Gender	
Female	25,400 (52.0%)
Male	23,416 (48.0%)
Health insurance coverage	
Public health insurance	5779 (11.8%)
Private health insurance	36,821 (75.4%)
Admission costs paid with patient’s own resources	6216 (13.7%)
Comorbidities	
Diabetes mellitus	11,037 (22.6%)
Cancer	9647 (19.8%)
Chronic renal failure	4469 (9.2%)
Coronary artery disease	2993 (6.1%)
Cardiac failure	2468 (5.1%)
Chronic pulmonary disease	2551 (5.2%)
Charlson Comorbidity Index (points)	1 (0–2)

Characteristics	*n* = 48,816
Functional status before hospital admission	
Ambulant	36,835 (75.5%)
Minor assistance	8556 (17.5%)
Major assistance or bedridden	3425 (7.0%)
Source of ICU admission	
Emergency department	25,738 (52.7%)
Operating room	14,979 (30.7%)
Ward/floor	4212 (8.6%)
Transfer from other hospitals	1876 (3.8%)
Other	2011 (4.1%)
Hospital days prior to ICU admission (*n*)	0 (0–1)
Admission diagnostic category	
Scheduled surgery	12,825 (26.3%)
Emergency surgery	2854 (5.8%)
Cardiovascular**	5511 (11.3%)
Sepsis**	10,876 (22.3%)
Neurological**	5588 (11.4%)
Respiratory**	2542 (5.2%)
Gastrointestinal**	2263 (4.6%)
Other medical admissions**	6357 (13.0%)
SAPS 3 (points)	43 (34–53)
SOFA score on Day 1 (points)	1 (0–4)
Support on Day 1	
Mechanical ventilation	7550 (15.5%)
Noninvasive ventilation	4875 (10.0%)
Vasopressors	6158 (12.6%)
Renal replacement therapy	1578 (3.2%)
ICU LOS (days)	3 (1–5)
Hospital LOS (days)	8 (4–18)
ICU mortality	5385 (11.0%)
Hospital mortality	8031 (16.5%)
Destination at hospital discharge	
Home	37,683 (77.2%)
Other hospital	556 (1.1%)
Hospice/home care	355 (0.7%)
Other/unknown	2191 (4.5%)
Died	8031 (16.5%)

Results for continuous variables are reported as mean ± SD and median (IQR)

*IQR* interquartile range, *ICU* intensive care unit, *SAPS* Simplified Acute Physiology Score, *SOFA* Sequential Organ Failure Score, *LOS* length of stay, *SD* standard deviation

** These admission categories refer to medical diagnosis only

Median ICU and hospital LOS were 3 (1–5) and 8 (4–18) days, respectively. Mean SAPS 3 was 44.3 ± 15.4 points. A total of 5385 (11.0%) died in the ICUs; 2646 died in the hospital after the ICUs discharge and the hospital mortality rate was 16.5%. Table 2 reports the main patients’ characteristics and outcomes.

**Fig. 2:**
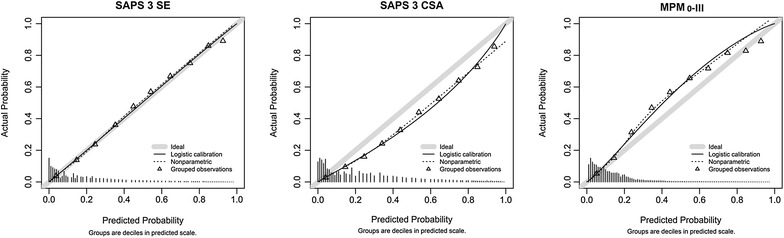
Calibration plots for SAPS 3-SE, SAPS 3-CSA and MPM_0_-III, with predicted mortality rates stratified by 10% intervals of mortality risk (*x*-axis) against observed mortality rates (*y*-axis)

**Fig. 3:**
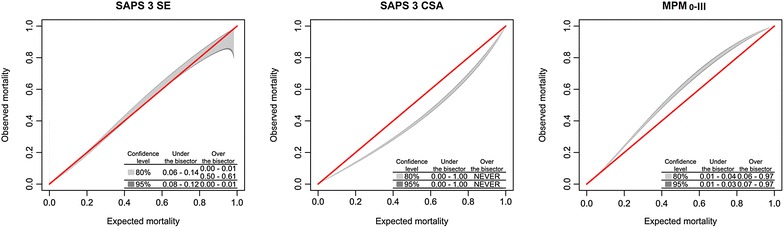
Calibration Belt for SAPS 3-SE, SAPS 3-CSA and MPM_0_-III, described as bisector deviation intervals, as proposed by GiViTI, (Italian Group for the Evaluation of Intervention in Intensive Care Medicine). The times the calibration belt significantly deviates from the bisector using 80 and 95% confidence levels are described in the lower right part of the *plots*

Predicted mortality rates were 16.4 ± 19.3% (SAPS 3-SE), 21.7 ± 23.2% (SAPS 3-CSA) and 14.3 ± 14.0% (MPM_0_-III). Table 3 gives the performance analyses for the studied scores. In summary, the SMR was appropriate using the SAPS 3-SE, while the SAPS 3-CSA overestimated and the MPM_0_-III underestimated the hospital mortality. Overall, discrimination was good, but higher for the SAPS 3 score (Table 3). Calibration was acceptable for the SAPS 3-SE only. In the calibration belt analysis, there was only minimal over- (below the first percentile) and underprediction (between the 8th and 14th percentiles) in the first two risk deciles. Conversely, the SAPS 3-CSA uniformly overestimated mortality in all risk range and the MPM_0_-III tended in general to underestimation (Figs. 2, 3).

**Table 3:** Scores performances comparison

	Mortality		Discrimination		Calibration*		Precision
	Predicted mortality (±SD)	SMR (95% CI)	AUROC	95% CI	Over the bisector 95% CI	Under the bisector 95% CI	Brier score
SAPS 3-SE	16.4 ± 19.3%	1.00 (0.98–1.02)	0.850	0.84–0.85	0.00–0.01	0.08–0.12	0.098
SAPS 3-CSA	21.7 ± 23.2%	0.75 (0.74–0.77)	0.850	0.84–0.85	Never	Always	0.103
MPM0-III	14.3 ± 14.0%	1.15 (1.13–1.18)	0.800	0.79–0.80	0.07–0.97	0.01–0.03	0.111

*SAPS 3-SE* Simplified Acute Physiology Score 3-Standard Equation, *SAPS 3-CSA* Simplified Acute Physiology Score 3-Customized equation for Central and South American Countries, *MPM0-III* Mortality Probability Models III, SMR Standardized mortality rates, *AUROC* area under the curve

* Calibration described as bisector deviation intervals, as proposed by GiViTI, (Italian Group for the Evaluation of Intervention in Intensive Care Medicine)

As most of the included ICUs were located at private hospitals, we performed subgroup analyses according to the type of hospital and specific subgroups of patients (Additional file 1: eTable 1 and eFigures 1–8). In patients admitted to private hospitals, we found results comparable to the ones observed for all the studied population and the SAPS 3-SE was the only model with a good performance. However, in patients admitted to public hospitals, none of the models was accurate in predicting hospital mortality. Finally, we performed additional analyses of the SAPS 3 performance in all patients (*n* = 55,742) fulfilling only the eligibility criteria reported the original publication of the model [4]. Models’ discrimination (AUROC = 0.855) for both the SAPS 3-SE and SAPS 3-CSA and calibration (Additional file 1: eFigure 3) were also appropriate. In Additional file 1: eTable 2, we provided information on patients’ characteristics and outcomes for our cohort of patients and the one reported in the SAPS 3 study.

## Discussion

In the present study, we demonstrated that the SAPS 3-SE was able to accurately predict outcomes in a large contemporary cohort of Brazilian ICU patients. Conversely, the MPM_0_-III score had a relatively worse calibration and tended to significantly underestimate mortality, while the SAPS 3-CSA overestimated mortality despite a reasonable discrimination. Moreover, the SAPS 3-SE provided more precise estimations, resulting in a SMR closer to 1.0. In the calibration curves, the lines of observed mortality of the SAPS 3-SE were uniformly closer to the line of ideal prediction across all risk classes.

In the last years, mostly driven by official recommendations provided by AMIB, the SAPS 3 became the severity-of-illness score used in the vast majority of Brazilian ICUs to evaluate ICU performance as well as for benchmarking. However, validation studies of SAPS 3 were performed in specific subgroup of patients or in single-center studies involving a general ICU population [7–16]. In general, both the SAPS 3-SE and SAPS 3-CSA equations were evaluated in the studies. Overall, discrimination was usually good, but calibration results varied among the studies.

In these previous studies, the SAPS 3-SE had a poor calibration and tended usually to underestimate mortality [7–10, 12]. The SAPS 3-SE tended to overestimate mortality in only two studies (one of them comprising patients with acute coronary syndromes), both with a relatively low mortality rate [11, 16]. On the other hand, the SAPS 3-CSA accurately predicted mortality in five studies involving patients with cancer [8, 9], acute kidney injury [10, 12] and those who underwent surgical procedures [15]. Our results confirm that the MPM_0_-III, however, was inaccurate in predicting mortality. These results are in line with almost all previous studies performed in Brazil [9, 10, 12, 16].

There is a known phenomenon with traditional calibration statistics (such as Hosmer–Lemeshow goodness of fit) in prediction models validation/calibration studies with many thousands included subjects, in which often p values are highly significant despite visually good calibration curves, very small absolute errors, and acceptable calibration slope and intercept. This occurs because with a large sample size the power is big enough to detect, as statistically significant, irrelevant small differences. At the other extreme, one must be cautious in the interpretation of calibration results with small cohorts, because, even when the calibration curve, the calibration intercept and slope points to a miscalibration, the p values of traditional calibration statistics may not be significant, raising concern about the study low power [25]. Therefore, in small cohorts, the lack of correspondence between expected and observed probabilities can also result in misaligned calibration curves, when sample size cannot be enough to achieve statistical significance [26]. In addition, specific subgroups of patients were included in these studies, whose results may not be fully transposed to general populations of critical care patients in different scenarios.

It is a well-known phenomenon that the performance of prognostic scores (chiefly the calibration) tends to deteriorate overtime. Zimmerman et al. [2] when reporting the development of the APACHE IV elegantly demonstrated this. Soares et al. [8] also documented the temporal compromising of calibration studying the SAPS 3 score in a cohort of patients with cancer admitted to the ICU over a 3-year period in Brazil. This is why the performance of prognostic scores should be reassessed periodically.

The cohort composition could also interfere with the score performance. Comparing our cohort and original SAPS 3 development cohort, we had comparable median age, but clinical patients predominated (67.9 vs. 43.5% in the SAPS 3 cohort), with lower median SAPS 3 scores (43 points vs. 48 points) and lower hospital mortality (16.5 vs. 23.5%) (Additional file 1: eTable 2). Despite these case mix differences, currently the SAPS 3-SE model was well fitted to our population, which might reflect changes in the provision of health care resulting in lower risk-adjusted mortality. In this sense, our results have potential implications for ICU performance evaluation and more importantly for benchmarking purposes in Brazilian ICUs. On the one hand, we provide robust evidence that although the SAPS 3 remains useful in our country, the customized equation for Latin American countries should be no longer used.

Our study has many strengths including being, to our knowledge, the largest validation study of severity-of-illness scores in Brazil and using more contemporary data from several centers countrywide. Moreover, we consider there is a negligible potential for discharge bias, [27] once our percentage of patients discharged to other hospitals and hospice care facilities was minimal.

Our study has also several limitations that should be considered in the interpretation of our results. First, although we have evaluated a large number of Brazilian ICUs, we used a convenience sample, predominantly composed by private hospitals and they may not be representative of the entire country. Second, we have not audited data collection, as we used data recorded in a registry for performance evaluation and benchmarking. Therefore, we cannot estimate the effect of missing variables in the scores estimations. However, trained healthcare professionals that work as case managers register data in all ICUs. Third, we did not assess end-of-life decisions, as they are not regularly registered in the database, and therefore, we were unable to account for this factor in the analysis.

In conclusion, using a large contemporary database, we demonstrated that the SAPS 3-SE was accurate in predicting outcomes, supporting its use for performance evaluation and benchmarking in Brazilian ICUs.

## Additional file

**Additional file 1.**Electronic Supplementary Material for External Validation of SAPS 3 and MPM0-III scores in 48,816 patients from 72 Brazilian ICUs.

## Abbreviations

AMIB: Associação de Medicina Intensiva Brasileira
APACHE: Acute Physiology and Chronic Health Evaluation
AUROC: area under receiver operating characteristic
BRICNet: Brazilian Research in Intensive Care Network
CI: confidence interval
ESM: electronic supplementary material
ICU: intensive care unit
LOS: length of stay
MPM0-III: Mortality Probability Model III
SAPS: Simplified Acute Physiology Score
SAPS 3-CSA: SAPS 3, customized equation for Central and South American Countries
SAPS 3-SE: SAPS 3, standard equation
SMR: standardized mortality rates
SOFA: sequential organ failure assessment
