# Supplementary material for: External validation of SAPS 3 and MPM0-III scores in 48,816 patients from 72 Brazilian ICUs
Source: Ann Intensive Care. 2017 May 18;7:53. doi: 10.1186/s13613-017-0276-3 (PMC5436994; doi:10.1186/s13613-017-0276-3)

**External Validation of SAPS 3 and MPM 0-III scores in 48,816 patients from 72 Brazilian ICUs**

Authors: Moralez et al. for the ORCHESTRA (ORganizational CHaractEeriSTics in cRitcal cAre) Study Investigators.

**Electronic Supplementary Material**

**Index**

**Information Page**

**List of Participating Centers 3**

**Supplementary Tables 7**

**Supplementary Figures 9**

**Investigators and Participating Centers**

***Steering Committee:*** Marcio Soares (PI), Jorge I. F. Salluh, Fernando A. Bozza (Instituto D’Or de Pesquisa e Ensino, Rio de Janeiro); Derek C. Angus, Jeremy M. Kahn (CHRISMA, University of Pittsburgh Medical Center, Pittsburgh)

***Data Management and Secretariat:*** Grazielle V. Ramos, Aline R. Silva (Instituto D’Or de Pesquisa e Ensino, Rio de Janeiro)

***Statistical analyses:*** Marcio Soares, Pedro E. A.A Brasil (Instituto D’Or de Pesquisa e Ensino, Rio de Janeiro)

| **Investigators** | | |  |  |  |
| --- | --- | --- | --- | --- | --- |
| **First Name** | **Last Name** | **Institution** | | **City** | **State** |
| Maristela | Machado | Hospital Agenor Paiva | | Salvador | Bahia |
| Josianne | Guimarães | Hospital Agenor Paiva | | Salvador | Bahia |
| Maria | Fauaze | Hospital Agenor Paiva | | Salvador | Bahia |
| Meton | Alencar | Hospital Regional do Cariri | | Juazeiro do Norte | Ceará |
| Ivo | Bonfim | Hospital Regional do Cariri | | Juazeiro do Norte | Ceará |
| Francisco | Souza | Hospital Regional do Cariri | | Juazeiro do Norte | Ceará |
| Rubens | Ribeiro | Hospital Anchieta | | Taguatinga | Distrito Federal |
| Rodrigo | Biondi | Hospital Anchieta | | Taguatinga | Distrito Federal |
| Raquel | Fonseca | Hospital Anchieta | | Taguatinga | Distrito Federal |
| Fábio | Amorim | Hospital Anchieta | | Taguatinga | Distrito Federal |
| Eduardo | Lessa | Hospital Anchieta | | Taguatinga | Distrito Federal |
| Auriane | Barcelar | Hospital Anchieta | | Taguatinga | Distrito Federal |
| Hélia | Araújo | Hospital do Coração do Brasil | | Brasília | Distrito Federal |
| Leandro | Goulart | Hospital do Coração do Brasil | | Brasília | Distrito Federal |
| Yara | Aguiar | Hospital do Coração do Brasil | | Brasília | Distrito Federal |
| Marcelo | Maia | Hospital Santa Luzia | | Brasília | Distrito Federal |
| Gisele | Maganan | Hospital Santa Luzia | | Brasília | Distrito Federal |
| Eliana | Caser | Hospital Unimed Vitória | | Vitória | Espírito Santo |
| Silvane | Damasceno | Hospital Unimed Vitória | | Vitória | Espírito Santo |
| Ana Paula | Moraes | Hospital Geral Tarquínio Lopes Filho | | São Luís | Maranhão |
| Alexandre | Carvalho | UDI Hospital | | São Luís | Maranhão |
| Ana Cláudia | Carvalho | UDI Hospital | | São Luís | Maranhão |
| Edilene | Novaes | UDI Hospital | | São Luís | Maranhão |
| Marcelo | Sousa | Santa Casa de Caridade de Diamantina | | Diamantina | Minas Gerais |
| José | Andrade | Santa Casa de Caridade de Diamantina | | Diamantina | Minas Gerais |
| Ana Luiza | Souza | Santa Casa de Caridade de Diamantina | | Diamantina | Minas Gerais |
| Marcia | Souza | Santa Casa de Caridade de Diamantina | | Diamantina | Minas Gerais |
| Guilherme | Fernandes | Santa Casa de Misericórdia de Juiz de Fora | | Juiz de Fora | Minas Gerais |
| Wilson | Neto | Santa Casa de Misericórdia de Juiz de Fora | | Juiz de Fora | Minas Gerais |
| Guilherme | Jacome | Santa Casa de Misericórdia de Juiz de Fora | | Juiz de Fora | Minas Gerais |
| Meire | Almeida | Santa Casa de Misericórdia de Juiz de Fora | | Juiz de Fora | Minas Gerais |
| Maria | Van Keulen | Santa Casa de Misericórdia de Juiz de Fora | | Juiz de Fora | Minas Gerais |
| Ciro | Mendes | Hospital Universitário Lauro Wanderley | | João Pessoa | Paraíba |
| Paulo | Gottardo | Hospital Universitário Lauro Wanderley | | João Pessoa | Paraíba |
| Mariza | Lima | Hospital Esperança | | Recife | Pernambuco |
| Marçal | Paiva | Hospital Esperança | | Recife | Pernambuco |
| Cristiane | Mendes | Hospital Esperança | | Recife | Pernambuco |
| Maria de Fátima | Mesquita | Hospital Esperança | | Recife | Pernambuco |
| Roberto | Burel | Hospital Esperança | | Recife | Pernambuco |
| Michele | Godoy | Hospital Prontolinda | | Olinda | Pernambuco |
| Gustavo | Rocha | Hospital Prontolinda | | Olinda | Pernambuco |
| Tatiana | Cadena | Hospital Prontolinda | | Olinda | Pernambuco |
| Leonardo | Diamante | Hospital Prontolinda | | Olinda | Pernambuco |
| Lanecley | Fulco | Hospital Prontolinda | | Olinda | Pernambuco |
| Jeanine | Guerra | Hospital Prontolinda | | Olinda | Pernambuco |
| Sérgio | Cavalcanti | Hospital São Marcos | | Recife | Pernambuco |
| Luciane | Ishy | Hospital São Marcos | | Recife | Pernambuco |
| João | Rodolfo | Hospital São Marcos | | Recife | Pernambuco |
| Weidson | Dantas | Hospital São Marcos | | Recife | Pernambuco |
| Bárbara | Souza | Hospital São Marcos | | Recife | Pernambuco |
| Sandro | Oliveira | Hospital Bangu | | Rio de Janeiro | Rio de Janeiro |
| Adriana | Campos | Hospital Bangu | | Rio de Janeiro | Rio de Janeiro |
| Walter | Homena Jr | Hospital Barra D’Or | | Rio de Janeiro | Rio de Janeiro |
| Francisco | Gabriel | Hospital Barra D’Or | | Rio de Janeiro | Rio de Janeiro |
| Marcelo | Santino | Hospital Barra D’Or | | Rio de Janeiro | Rio de Janeiro |
| Diamantino | Salgado | Hospital Barra D’Or | | Rio de Janeiro | Rio de Janeiro |
| Carlos | André | Hospital Barra D’Or | | Rio de Janeiro | Rio de Janeiro |
| William | Viana | Hospital Copa D’Or | | Rio de Janeiro | Rio de Janeiro |
| Cecilia | Magno | Hospital Copa D’Or | | Rio de Janeiro | Rio de Janeiro |
| Marco Aurélio | Fernandes | Hospital Copa D’Or | | Rio de Janeiro | Rio de Janeiro |
| Álvaro | Pontes | Hospital Copa D’Or | | Rio de Janeiro | Rio de Janeiro |
| Maria Eduarda | Tavares | Hospital Copa D’Or | | Rio de Janeiro | Rio de Janeiro |
| Felipe | Saddy | Hospital Copa D’Or | | Rio de Janeiro | Rio de Janeiro |
| Enio | Schroedder | Hospital Copa D’Or | | Rio de Janeiro | Rio de Janeiro |
| Marcelo | Lugarinho | Hospital de Clínicas Mario Lioni | | Duque de Caxias | Rio de Janeiro |
| Moyzes | Damasceno | Hospital de Clínicas de Niterói | | Niterói | Rio de Janeiro |
| Felipe | Ribeiro | Hospital de Clínicas de Niterói | | Niterói | Rio de Janeiro |
| Diogo | Terrana | Hospital de Clínicas de Niterói | | Niterói | Rio de Janeiro |
| Valdênia | Souza | Hospital de Clínicas de Niterói | | Niterói | Rio de Janeiro |
| Angelo | di Candia | Hospital de Clínicas de Niterói | | Niterói | Rio de Janeiro |
| Rodolfo | Spinoza | Hospital do Câncer II – Instituto Nacional de Câncer | | Rio de Janeiro | Rio de Janeiro |
| José | Netto | Hospital do Câncer II – Instituto Nacional de Câncer | | Rio de Janeiro | Rio de Janeiro |
| Alexandre | Scotti | Hospital Israelita Albert Sabin | | Rio de Janeiro | Rio de Janeiro |
| Roberto | Lannes | Hospital Municipal Souza Aguiar | | Rio de Janeiro | Rio de Janeiro |
| Sion | Filho | Hospital Municipal Souza Aguiar | | Rio de Janeiro | Rio de Janeiro |
| Andrea | Ludovico | Hospital Municipal Souza Aguiar | | Rio de Janeiro | Rio de Janeiro |
| Jorge | Pinto | Hospital Norte D’Or | | Rio de Janeiro | Rio de Janeiro |
| Márcia | Menezes | Hospital Oeste D’Or | | Rio de Janeiro | Rio de Janeiro |
| Rosa | Stancato | Hospital Oeste D’Or | | Rio de Janeiro | Rio de Janeiro |
| Carolina | Braga | Hospital Oeste D’Or | | Rio de Janeiro | Rio de Janeiro |
| Joyce | Andrade | Hospital Oeste D’Or | | Rio de Janeiro | Rio de Janeiro |
| Guilherme | Faria | Hospital Oeste D’Or | | Rio de Janeiro | Rio de Janeiro |
| Liliane | Mendonça | Hospital Oeste D’Or | | Rio de Janeiro | Rio de Janeiro |
| Alcino | Toledo | Hospital Oeste D’Or | | Rio de Janeiro | Rio de Janeiro |
| Pedro | Varaschin | Hospital Pasteur | | Rio de Janeiro | Rio de Janeiro |
| Renata | Orofino | Hospital Pasteur | | Rio de Janeiro | Rio de Janeiro |
| Anna | Mundt | Hospital Pasteur | | Rio de Janeiro | Rio de Janeiro |
| Roberto | Costa | Hospital Quinta D’Or | | Rio de Janeiro | Rio de Janeiro |
| Cristiane | Belo | Hospital Quinta D’Or | | Rio de Janeiro | Rio de Janeiro |
| Eduardo | Xavier | Hospital Quinta D’Or | | Rio de Janeiro | Rio de Janeiro |
| Márcio | Guimarães | Hospital Quinta D’Or | | Rio de Janeiro | Rio de Janeiro |
| Cristiane | Oliveira | Hospital Quinta D’Or | | Rio de Janeiro | Rio de Janeiro |
| Juliana | Silveira | Hospital Quinta D’Or | | Rio de Janeiro | Rio de Janeiro |
| Leonardo | Campioni | Hospital Quinta D’Or | | Rio de Janeiro | Rio de Janeiro |
| Alessandra | Longo | Hospital Quinta D’Or | | Rio de Janeiro | Rio de Janeiro |
| Alessandra | Alves | Hospital Rios D’Or | | Rio de Janeiro | Rio de Janeiro |
| Ricardo | Lima | Hospital Samaritano | | Rio de Janeiro | Rio de Janeiro |
| Marcos | Knibel | Hospital São Lucas Copacabana | | Rio de Janeiro | Rio de Janeiro |
| Pedro | Azambuja | Hospital São Lucas Copacabana | | Rio de Janeiro | Rio de Janeiro |
| Christian | Roderjan | Hospital São Lucas Copacabana | | Rio de Janeiro | Rio de Janeiro |
| Rodrigo | Hatum | Hospital Total Cor | | Rio de Janeiro | Rio de Janeiro |
| André | Japiassú | Hospital Total Cor | | Rio de Janeiro | Rio de Janeiro |
| Patrícia | D’Alessandro | Clínica São Vicente | | Rio de Janeiro | Rio de Janeiro |
| Arthur | Vianna | Clínica São Vicente | | Rio de Janeiro | Rio de Janeiro |
| Denise | Medeiros | Centro Hospitalar do Instituto de Pesquisa Clínica Evandro Chagas – Instituto Nacional de Infectologia - Fiocruz | | Rio de Janeiro | Rio de Janeiro |
| André | Japiassú | Centro Hospitalar do Instituto de Pesquisa Clínica Evandro Chagas – Instituto Nacional de Infectologia - Fiocruz | | Rio de Janeiro | Rio de Janeiro |
| Ulysses | Silva | Fundação Pio XII – Hospital de Câncer de Barretos | | Barretos | São Paulo |
| Fernando | Colombari | Hospital Alemão Oswaldo Cruz | | São Paulo | São Paulo |
| Alexandre | Cavalcanti | Hospital do Coração | | São Paulo | São Paulo |
| Edson | Romano | Hospital do Coração | | São Paulo | São Paulo |
| Rosianne | Vasconcelos | Hospital do Coração | | São Paulo | São Paulo |
| Suzana | Lobo | Hospital de Base de São José do Rio Preto | | São José do Rio Preto | São Paulo |
| Thiago | Corrêa | Hospital Israelita Albert Einstein | | São Paulo | São Paulo |
| Eliezer | Silva | Hospital Israelita Albert Einstein | | São Paulo | São Paulo |
| Alexandre | Maciel | Hospital São Camilo Pompéia | | São Paulo | São Paulo |
| Marcus | Ferez | Hospital São Francisco | | Ribeirão Preto | São Paulo |
| Edson | Nicolini | Hospital São Francisco | | Ribeirão Preto | São Paulo |
| Brunno | Silva | Hospital São Francisco | | Ribeirão Preto | São Paulo |
| Kamila | Iazzetta | Hospital São Francisco | | Ribeirão Preto | São Paulo |
| André | Nunes | Hospital São Luiz – Unidade Anália Franco | | São Paulo | São Paulo |
| Rafaela | Morsch | Hospital São Luiz – Unidade Anália Franco | | São Paulo | São Paulo |
| Haggeas | Fernandes | Hospital São Luiza – Unidade Brasil | | Santo André | São Paulo |
| Leonardo | Brauer | Hospital São Luiz – Unidade Itaim | | São Paulo | São Paulo |
| Bruno | Mazza | Hospital São Luiz – Unidade Jabaquara | | São Paulo | São Paulo |
| Débora | Mazza | Hospital São Luiz – Unidade Jabaquara | | São Paulo | São Paulo |
| Roberto | Filho | Hospital São Luiz – Unidade Jabaquara | | São Paulo | São Paulo |
| Bruno | Mazza | Hospital São Luiz – Unidade Morumbi | | São Paulo | São Paulo |
| Débora | Mazza | Hospital São Luiz – Unidade Morumbi | | São Paulo | São Paulo |
| Luciano | Azevedo | Hospital Sírio-Libanês | | São Paulo | São Paulo |
| Guilherme | Schettino | Hospital Sírio-Libanês | | São Paulo | São Paulo |
| Fernando | Marco | Hospital viValle | | São José dos Campos | São Paulo |
| Guilherme | Barros | Hospital viValle | | São José dos Campos | São Paulo |
| Thiago | Lisboa | Santa Casa de Porto Alegre – Hospital Dom Vicente Scherer | | Porto Alegre | Rio Grande do Sul |
| Edison | Filho | Santa Casa de Porto Alegre – Hospital Dom Vicente Scherer | | Porto Alegre | Rio Grande do Sul |
| Thiago | Lisboa | Santa Casa de Porto Alegre – Hospital Santa Rita | | Porto Alegre | Rio Grande do Sul |
| André | Torelly | Santa Casa de Porto Alegre – Hospital Santa Rita | | Porto Alegre | Rio Grande do Sul |
| Thiago | Lisboa | Santa Casa de Porto Alegre – Pavilhão Central | | Porto Alegre | Rio Grande do Sul |
| Jorge | Hoher | Santa Casa de Porto Alegre – Pavilhão Central | | Porto Alegre | Rio Grande do Sul |
| Thiago | Lisboa | Santa Casa de Porto Alegre – Pavilhão Pereira Filho | | Porto Alegre | Rio Grande do Sul |
| Paula | Berto | Santa Casa de Porto Alegre – Pavilhão Pereira Filho | | Porto Alegre | Rio Grande do Sul |
| Jéssica | Oliveira | Santa Casa de Porto Alegre – Pavilhão Pereira Filho | | Porto Alegre | Rio Grande do Sul |

Supplementary Tables

eTable 1: SAPS 3 and MPM_0_-III scores performances, according health care providers (n=48,816)

SAPS 3- SE, Simplified Acute Physiology Score 3- Standard Equation, SAPS 3-CSA, Simplified Acute Physiology Score 3- Customized equation for Central and South American Countries, MPM_0_-III: Mortality Probability Models III, SMR, Standardized mortality rates; AUROC, area under the receiver operating characteristic curve

|  |  | SAPS 3-SE | SAPS 3-CSA | MPM_0_-III |
| --- | --- | --- | --- | --- |
|  |  |  |  |  |
| SMR (95%CI) | Overall | 1.00 (0.98-1.02) | 0.75 (0.74-0.77) | 1.15 (1.13-1.17) |
|  | Private | 0.91 (0.90-0.93) | 0.68 (0.67-0.70) | 1.02 (1.00-1.04) |
|  | Public | 1.42 (1.39-1.46) | 1.13 (1.10-1.16) | 2.00 (1.95-2.05) |
| AUROC | Overall | 0.850 | 0.850 | 0.800 |
|  | Private | 0.850 | 0.850 | 0.809 |
|  | Public | 0.822 | 0.822 | 0.765 |
| Brier score | Overall | 0.098 | 0.103 | 0.111 |
|  | Private | 0.089 | 0.095 | 0.098 |
|  | Public | 0.180 | 0.169 | 0.228 |

eTable 2: Comparisons among demographic characteristics and main outcomes measures of patients from present study and from original SAPS 3 study.

|  | Brazilian validation cohort (patients fulfilling both  the SAPS 3 and MPM_0_-III eligibility criteria) | Brazilian validation cohort (patients fulfilling only SAPS 3 eligibility criteria) | Original SAPS 3  Hospital outcome cohort (1) |
| --- | --- | --- | --- |
| Patients (n) | 48,816 | 55,742 | 16,784 |
| SAPS 3, points (median, IQR) | 43 (34-53) | 45 (33-52) | 48 (38-69) |
| Age, years (median, IQR) | 65 (48-78) | 64 (48-78) | 64 (49-74) |
| Gender (n, %) |  |  |  |
| Male | 23,416 (48.0%) | 27,670 (49.6%) | 10,161 (60.5) |
| Female | 25,416 (52.0%) | 28,072 (50.4%) | 6610 (39.4) |
| Surgical/medical status (n, %) |  |  |  |
| Medical | 33,137 (67.9%) | 37,010 (66.4%) | 7305 (43.5) |
| Elective surgery | 12,825 (26.3%) | 15,646 (28.0%) | 5700 (34) |
| Emergency surgery | 2843 (5.8%) | 3086 (5.6%) | 2930 (17.5) |
| Missing | 0 | 0 | 849 (5.1) |
| ICU lenght of stay, days (median, IQR) | 3 (1-5) | 2 (1-5) | 2 (1-6) |
| ICU mortality (n, %) | 5383 (11.0)% | 5556 (10.0)% | 17.7% |
| Hospital mortality (n, %) | 8031 (16.5%) | 8371 (15.0%) | 23.5% |

IQR = interquartile range; ICU = intensive care unit; SAPS = Simplified Acute Physiology Score; MPM_0_-III: Mortality Probability Models III

1, Metnitz PGH, Moreno RP, Almeida E, Jordan B, Bauer P, Campos RA, et al. SAPS 3--From evaluation of the patient to evaluation of the intensive care unit. Part 1: Objectives, methods and cohort description. Intensive Care Med. 2005 Oct;31(10):1336–44

Supplementary Figures

eFigure 1:

Calibration curves of SAPS 3 and MPM_0_-III Scores - Private Healthcare Patients (n=43,962)

Calibration Plots for SAPS 3-SE, SAPS 3-CSA and MPM_0_-III, with predicted mortality rates stratified by 10% intervals of mortality risk (x-axis) against observed mortality rates (y-axis)

Calibration Belt for SAPS 3-SE, SAPS 3-CSA and MPM_0_-III, described as bisector deviation intervals, as proposed by GiViTI, (Italian Group for the Evaluation of Intervention in Intensive Care Medicine)

The times the calibration belt significantly deviates from the bisector using 80% and 95% confidence levels are described in the lower right part of the plots.


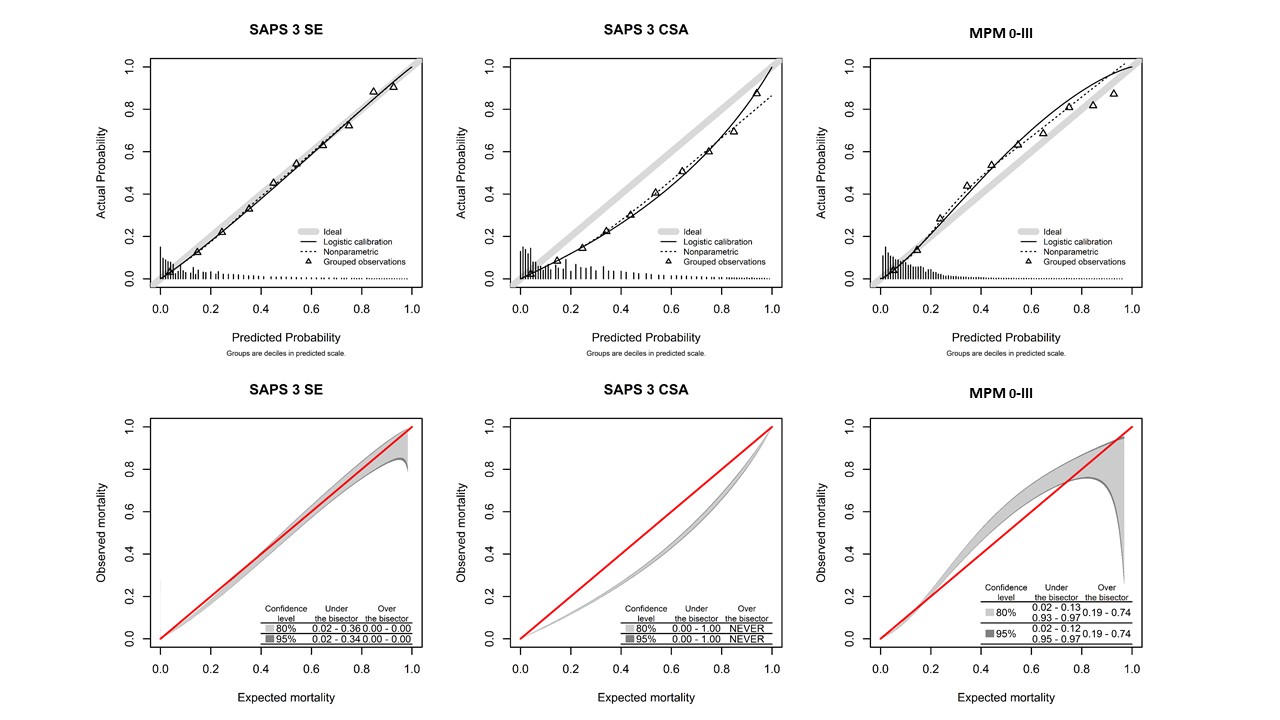


eFigure 2:

Calibration curves of SAPS 3 and MPM_0_-III Scores - Public Healthcare Patients (n=4854)

Calibration Plots for SAPS 3-SE, SAPS 3-CSA and MPM_0_-III, with predicted mortality rates stratified by 10% intervals of mortality risk (x-axis) against observed mortality rates (y-axis)

Calibration Belt for SAPS 3-SE, SAPS 3-CSA and MPM_0_-III, described as bisector deviation intervals, as proposed by GiViTI, (Italian Group for the Evaluation of Intervention in Intensive Care Medicine)

The times the calibration belt significantly deviates from the bisector using 80% and 95% confidence levels are described in the lower right part of the plots.


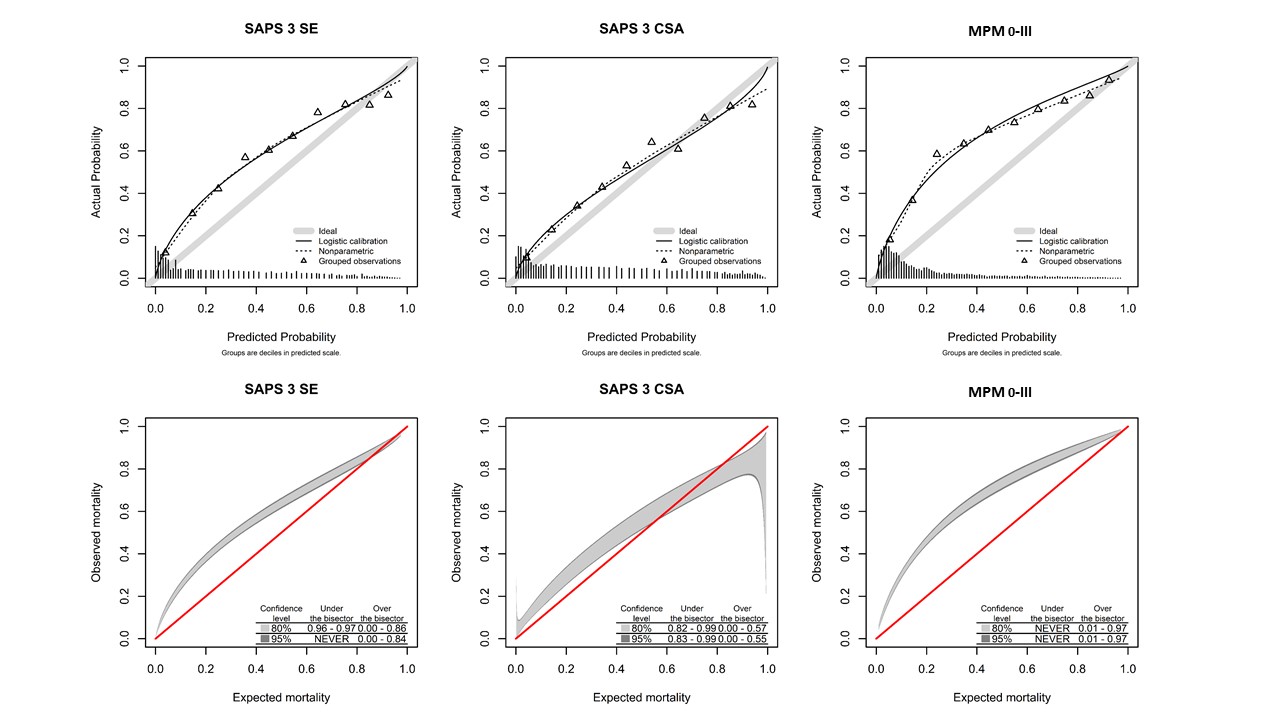


eFigure 3: SAPS 3 calibration curves, including all patients fulfilling only the SAPS 3 eligibility criteria (n=55,742)

Calibration Plots for SAPS 3-SE and SAPS 3-CSA, with predicted mortality rates stratified by 10% intervals of mortality risk (x-axis) against observed mortality rates (y-axis)

Calibration Belt for SAPS 3-SE and SAPS 3-CSA, described as bisector deviation intervals, as proposed by GiViTI, (Italian Group for the Evaluation of Intervention in Intensive Care Medicine)

The times the calibration belt significantly deviates from the bisector using 80% and 95% confidence levels are described in the lower right part of the plots.


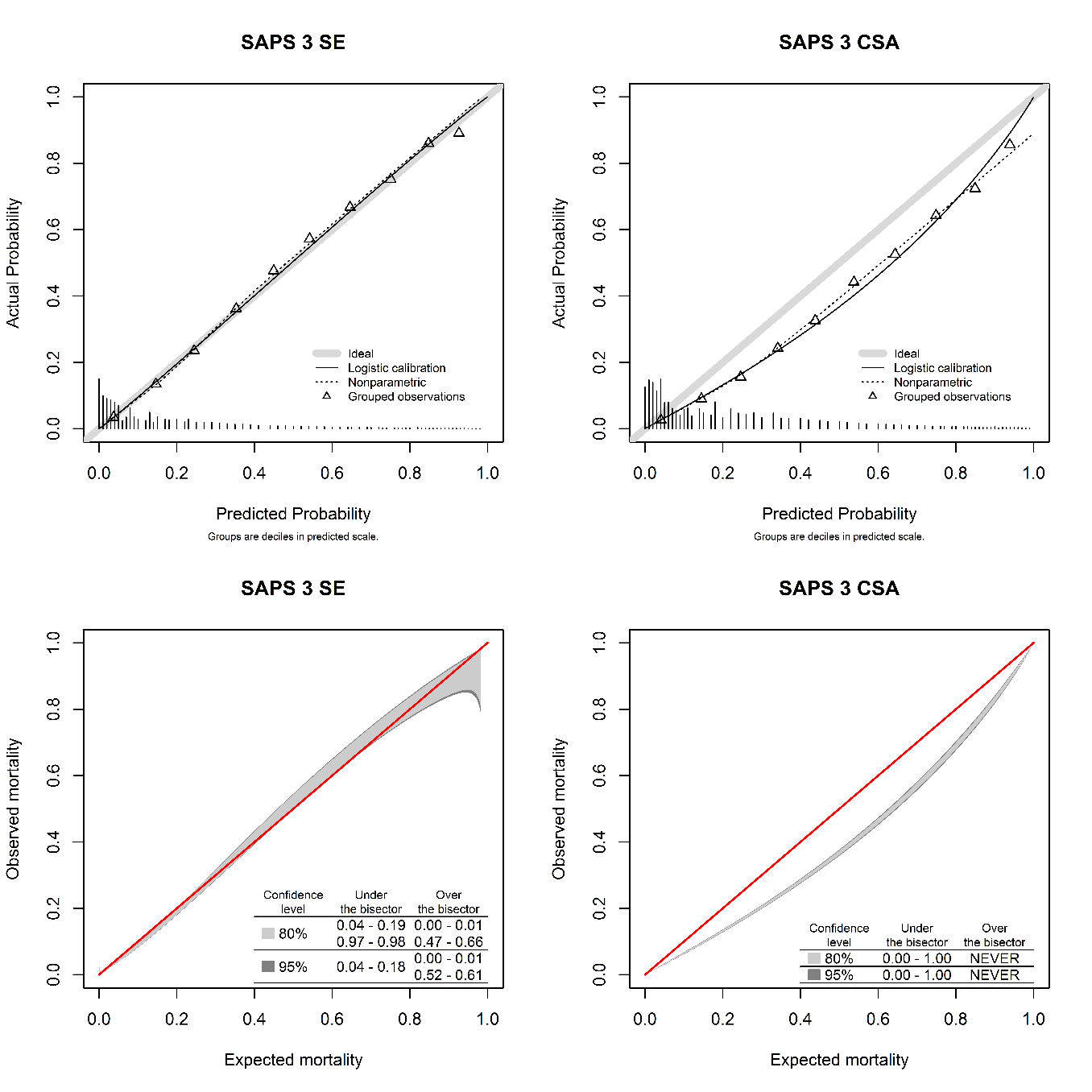


eFigure 4: SAPS 3- CSA subgroup analysis (n=48,816)


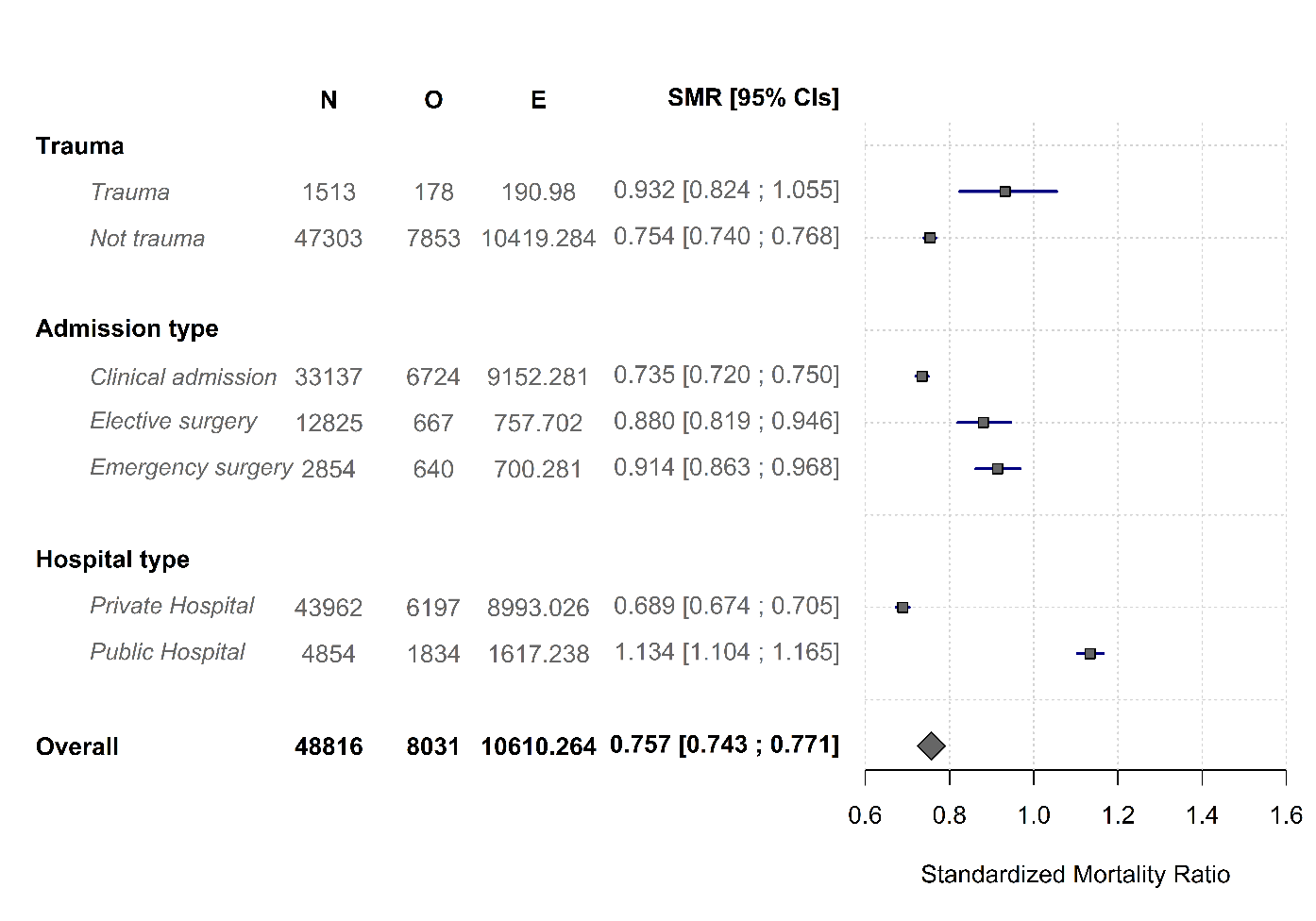


eFigure 5: SAPS 3- CSA subgroup analysis (n=55,742)


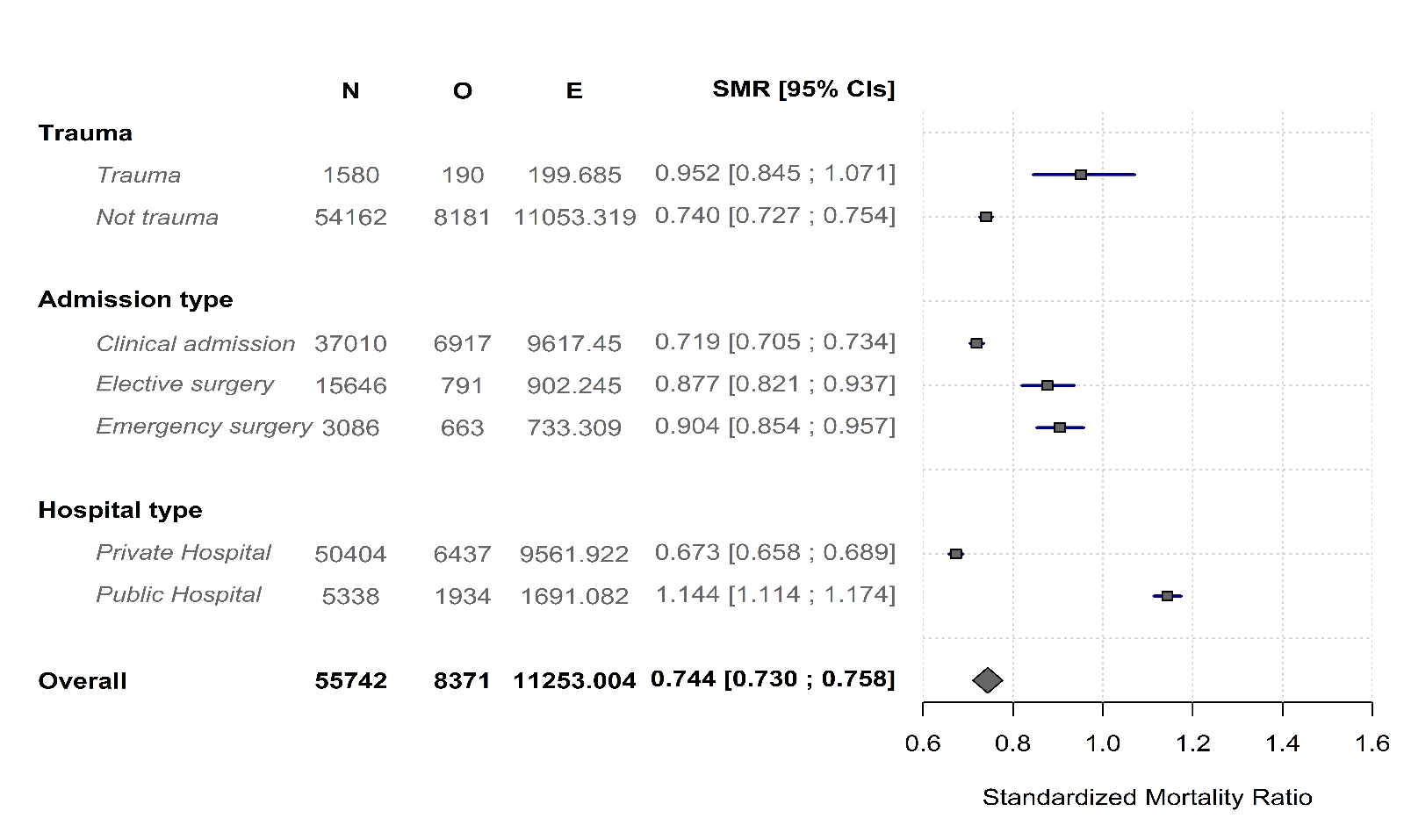


eFigure 6: SAPS 3-SE subgroup analysis (n=48,816)
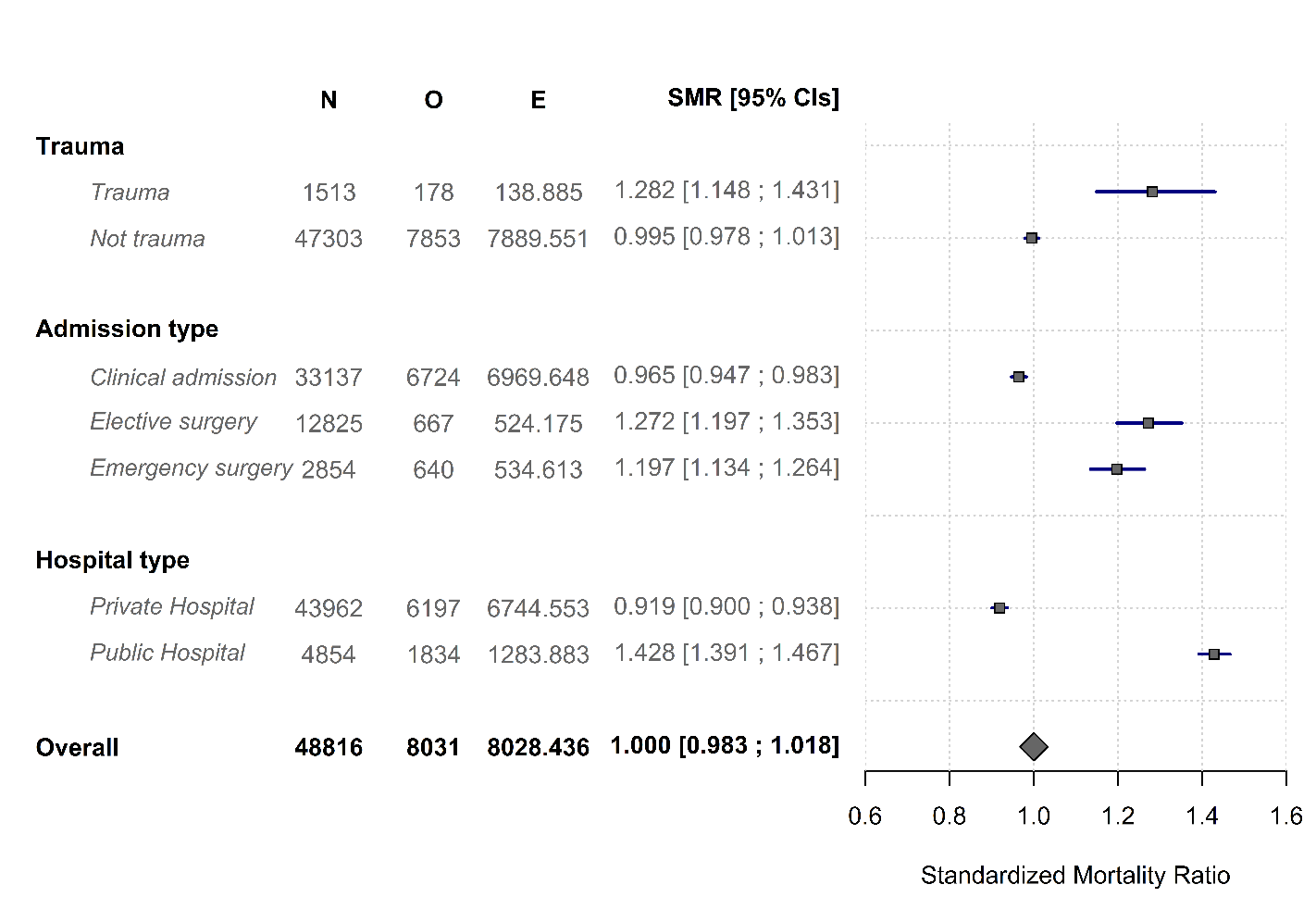


eFigure 7: SAPS 3- SE subgroup analysis (n=55,742)


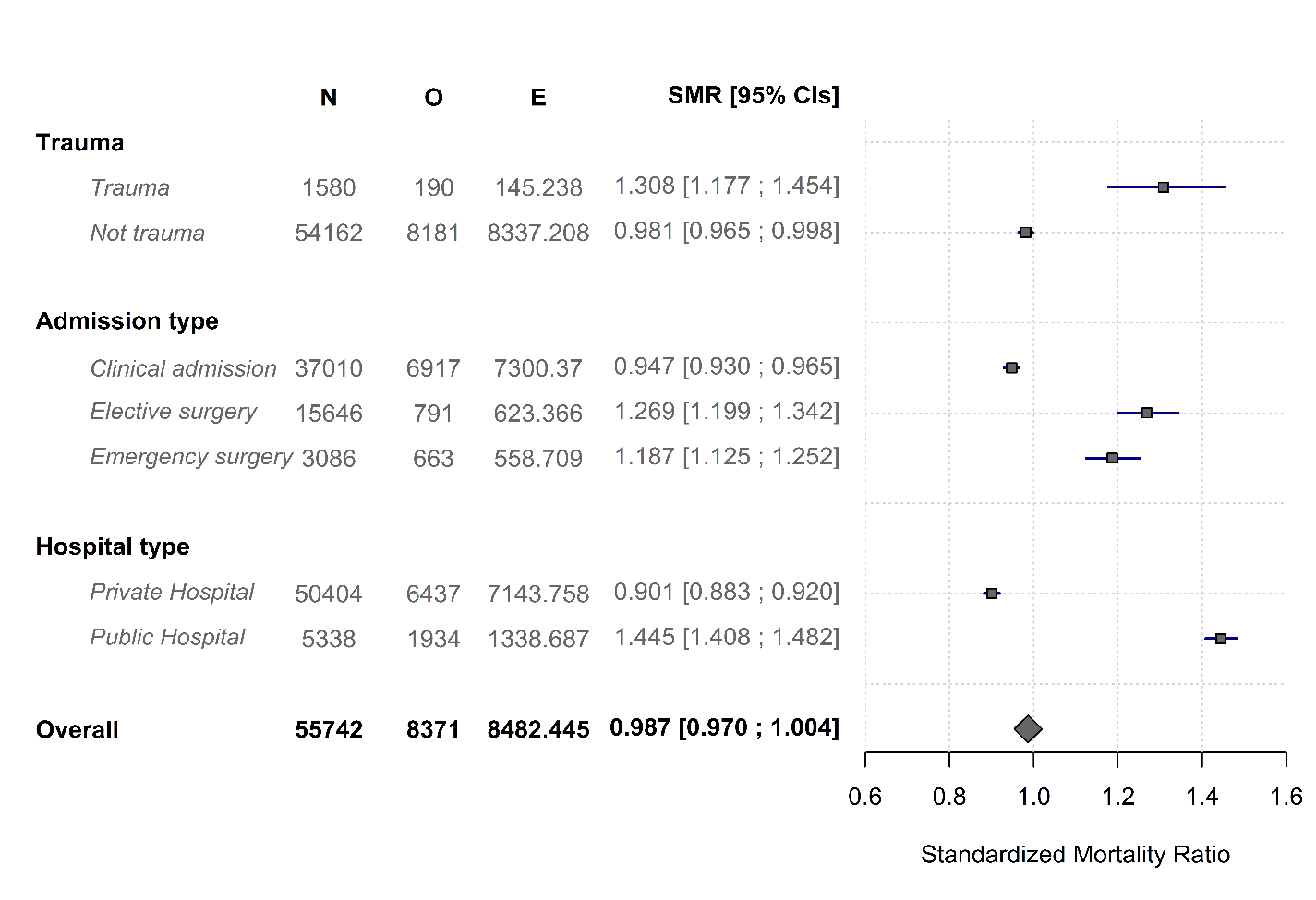


eFigure 8: MPM0-III subgroup analysis (n=48,816)


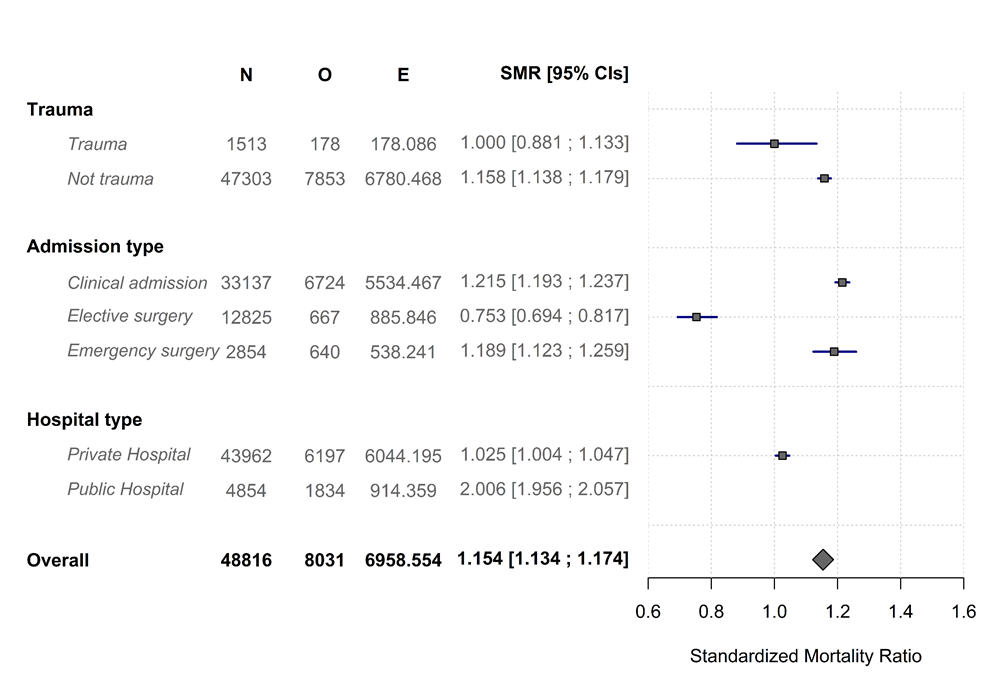

Supplement: Supplementary file 1 — Additional file 1.Electronic Supplementary Material for External Validation of SAPS 3 and MPM0-III scores in 48,816 patients from 72 Brazilian ICUs. [file 13613_2017_276_MOESM1_ESM.docx]
